# Sports, Myocarditis and COVID-19: Diagnostics, Prevention and Return-to-play Strategies

**DOI:** 10.1055/a-1810-5314

**Published:** 2022-06-03

**Authors:** Thomas Schmidt, Birna Bjarnason-Wehrens, Jonas Zacher, Hans-Georg Predel, Nils Reiss

**Affiliations:** 1 Institute for Cardiovascular Research, Schüchtermann-Klinik Bad Rothenfelde, Bad Rothenfelde, Germany; 2 Department of Preventive and Rehabilitative Sport and Exercise Medicine, Institute for Cardiology and Sports Medicine, German Sport University Cologne, Cologne, Germany

**Keywords:** sport, myocarditis, COVID-19, diagnostics, prevention, return-to-play

## Abstract

Myocarditis is an umbrella term for non-ischemic myocardial inflammation and remains a leading cause of sudden cardiac death in active individuals and athletes. Accurate diagnosing is challenging and diseases could often remain undetected. In the majority of cases, acute myocarditis resolves favourably. However, a relevant proportion of patients may have an increased risk of prognostically relevant cardiac arrhythmias and/or the development and progression of maladaptive myocardial remodelling (dilated cardiomyopathy). This review provides current knowledge on myocarditis and sports with special regard to the COVID-19 pandemic. Possible causes, common symptoms and proposed diagnostics are summarized. The relevance of temporary avoidance of intensive sports activities for both the prevention and therapy of acute myocarditis is discussed. Risk stratification, specific return-to-play recommendations and proposed follow-up diagnostics (also after COVID-19 infection) are presented.

## Introduction

Myocarditis (MC) is the third most frequent cause of sudden cardiac death (SCD) during physical activity in young sportsmen and women (≤35 years) in Germany 1 , and even at rest can trigger malignant arrhythmias 2 . The exact incidence of acute MC diseases is unclear since non-diagnosed and/or asymptomatic disease courses make it difficult to compile valid statistics 3 4 .

MC is an umbrella term for non-ischaemic myocardial inflammation, which can vary widely regarding symptoms, course, and prognosis 5 6 . Initially there is a short acute phase, during which the pathogens responsible for the inflammation reach the myocardium, negatively impact the heart cells and trigger an immune reaction. In the sub-acute phase, myocardial necrosis and fibrosis can then occur. Over the chronic course, MC predominantly resolves, and in most cases the inflammation subsides. Sometimes, however, small local non-ischaemic myocardial scars remain which can have an arrhythmogenic impact 7 8 9 .

Up to 20% of patients develop dilated cardiomyopathy (DCM) over the long-term course, sometimes taking years to become clinically evident 4 5 7 8 10 11 12 13 . The main dangers associated with MC are reduced systolic function of the myocardium, accompanied by comprehensive malperfusion of the organism, as well as an increased susceptibility to malignant arrhythmias and SCD 6 8 .

### Causes

The causes of MC are manifold 12 and can be categorised as follows:

- infectious (e. g. through viruses, bacteria, fungi, or parasites)
- toxic (e. g. through drug consumption, heavy metals, or radiation) and
- autoimmune (e. g. through rheumatic diseases, vaccination reactions, or medication intolerance).

In Europe and North America, MC is chiefly attributable to viral pathogens, such as a cold, influenza, or gastroenteritis 14 ; but bacterial infections, such as tonsillitis, scarlet fever, or borreliosis can also be the cause 8 .

In young patients and/or patients with sporting ambitions, it is also conceivable that drugs or doping agents are involved. Likewise, genetic predispositions can promote development of the disease 5 14 . In many MC patients it is ultimately impossible to determine the exact aetiology of the disease at a later stage. This can also be because no link is made between the cardiac problems occurring (often with a delay of days or even weeks) and a previous (seemingly harmless) infection 14 .

### Symptoms and diagnostics

Diagnosing MC is complex owing to its often heterogeneous course. Other cardiovascular diseases, such as coronary artery disease (CAD) or valvular vitia, must be excluded using differential diagnostics. The fact that sometimes (particularly in women) symptoms are only mild/modified should also be taken into account 5 . The following examinations can be used to diagnose MC and produce a clearer picture when taken as a synopsis 12 14 :

- medical history/symptoms
- electrocardiography (ECG)
- transthoracic echocardiography (TTE)
- biomarkers and/or inflammatory markers
- cardiovascular magnetic resonance imaging (CMR)
- endomyocardial biopsy (EMB).

#### Medical history/symptoms

Possible symptoms of MC can vary considerably in their manifestation. Degrees of severity range from a complete lack of symptoms to cardiac decompensation, cardiogenic shock or SCD 12 .

Frequently, chest pain and/or classic symptoms of heart failure (dyspnoea, performance drop) or arrhythmias (palpitations, dizziness, syncope) are initially described 14 . In a study including 670 cases of suspected MC, chest pain was the most common symptom, at 52% 15 . In the ITAMY study (n=386) 16 , 95% of MC patients with preserved left ventricular ejection fraction (LVEF>50%) had chest pain symptoms.

Athletic patients additionally report restricted physical performance, increased muscle soreness, as well as a slightly elevated heart rate both at rest and during exercise (approx. 5–10 bpm). However, in a differential diagnosis these symptoms can also point to “overtraining syndrome”, and this needs to be excluded 3 17 .

If the MC has an autoimmune cause, extra-cardiac symptoms can also occur (e. g. in conjunction with sarcoidosis or systemic sclerosis) and provide an indication of the underlying disease 14 .

#### ECG

In 42% of patients with suspected MC (96% in the ITAMY study) the resting ECG was conspicuous 15 16 . Non-specific changes which can occur include 6 15 :

- ventricular and supraventricular arrhythmias
- ST-segment deviations
- T-wave inversions
- conduction disorders
- low voltage.

In many patients, however, no special changes are discovered in the resting ECG. In elite endurance athletes, interpretation of the ECG signal can also prove difficult since similar ECG changes can occur as typical and non-pathological adaptations of the “athlete’s heart”. If available, the findings should therefore be compared to previous examinations in order to verify any changes. A 24-hour Holter ECG can be considered. The monitored time period should then also include a regular workout 3 .

#### TTE

Imaging with TTE is a standard diagnostic procedure. The following phenomena can provide indications of MC 3 6 7 :

- pericardial effusion
- left ventricular dilatation with thin myocardial walls
- increase in myocardial wall thickness (due to myocardial oedema)
- global or regional altered systolic function and wall motion abnormalities
- diastolic dysfunction.

Left ventricular ejection fraction (LVEF) can be slightly or considerably reduced at rest, but not necessarily 6 . In endurance athletes, the differentiation to physiological changes of athlete’s heart can be difficult. In these cases, previous findings should be taken as comparative images. A TTE can also be performed in a semi-recumbent position on a bicycle ergometer in order to be able to evaluate global systolic function and possible regional wall motion abnormalities during exercise. In diseased athletes, the wall motion abnormalities usually increase during physical exertion. In healthy athletes, the systolic function increases significantly during exercise 7 .

#### Biomarkers and/or inflammatory markers

In case of suspected MC, the following laboratory values are relevant 14 :

- cardiac troponin T/I
- C-reactive protein (CRP)
- creatine kinase (CK), creatine kinase-MB (CK-MB) and
- leukocytes.

These laboratory values are not specific MC markers, so that corresponding concentration increases can also occur with other diseases or non-pathological states. Nevertheless, the troponin T/I value in particular has proved helpful. In approx. 63% of all cases of suspected MC (100% in the ITAMY study), increased troponin values can be found 9 15 . MC is thus the second most frequent reason (after myocardial infarction) for an increased troponin value in patients below the age of 50 18 . The time factor plays a crucial role here: particularly in the initial phase following the first occurrence of symptoms (<1 month), increased values can be observed which can normalise again over the later course 14 .

In athletes it should be taken into account that corresponding biomarkers can also be physiologically increased following intense physical exercise and, at least temporarily, be beyond the threshold range. However, the increase in troponin caused by exercise is not quite as high and usually normalises again within 48 h. A sports anamnesis and repeat tests can provide the necessary information 3 7 .

#### CMR

CMR has become established in the last few years as one of the primary non-invasive diagnostic tools for patients with suspected MC 14 . Imaging provides information about global systolic function, local wall motion abnormalities, as well as a qualitative presentation of the tissue by visualising oedemas and fibroses 14 . Use of contrast medium and interpretation of a possible “late gadolinium enhancement” (LGE) have proved helpful 15 19 .

The updated Lake Louise Criteria cite as the main criteria for radiological proof of MC 19 :

- myocardial oedema (T2-weighted, T2-mapping) and/or
- myocardial injury (T1-weighted, T1-mapping, expanded extracellular volume, LGE).

Secondary criteria focus on pericardial effusion and left ventricular dysfunction 19 .

In the current discussion, the valency and sensitivity of CMR in the chronic phase of MC are controversial. For example, LGE is unable to differentiate clearly whether the inflammation/scar is fresh, ongoing or already healed. Estimating a patient’s sporting capability using this parameter is therefore difficult. However, studies show the prognostic significance of positive LGE for a major adverse cardiac event (MACE) 6 15 16 .

#### EMB

EMB is the gold standard among diagnostic examinations for acute MC, and yet it is not used routinely owing to its invasive nature 6 . It is used when standard treatment is not successful and the genesis of the MC is highly significant for the treatment 13 14 . With EMB, a distinction can be made between different pathogens using histological, immunohistological and viral polymerase chain reaction tests. In order to minimise potential false-negative findings, usually several (≥5–7) tissue samples of sufficient size (1–2 mm) are extracted from different cardiac areas 7 12 20 .

### Prevention, impact of sport, and preventive training breaks

#### Prevention and impact of sport

The risk of contracting MC can be reduced by protection from/minimisation of pathogen triggering (e. g. viral, bacterial, toxic, parasitic). The use of suitable protective clothing and an adequate level of vaccination appropriate to the country of residence is recommended 3 . Triggering factors also include drug and doping agent abuse, the significance of which should be explained within the framework of primary prophylaxis 3 14 .

Exposure to pathogens is not always avoidable. It is therefore crucial that the body’s own immune system is functioning. With regard to intensive sports activities, the following two problems arise in conjunction with the emergence of MC 14 21 :

- an assumed higher susceptibility to infection following intensive exercising (“open window effect”)
- a stronger MC development following intensive exercising in conjunction with an already existing infection.

It is generally assumed that regular moderate physical training induces multi-layer protective health effects and is concomitant with a stronger immune system 7 22 . However, blood test results show a temporarily reduced activation of the immune system following intensive physical exercise 23 . This phase can last for several hours and is known as the “open window effect”. It is assumed that pathogens can attack the organism more easily during this period 14 23 , but the significance of the “open window effect” is the subject of controversial debate 22 24 .

In professional athletes, the negative impact on the immune system of additive factors should not be underestimated, e. g. increased travelling, time differences, lack of sleep, extreme ambient conditions, depression or an insufficient time for regeneration 3 .

It is also assumed – in cases where an infection already exists – that intensive physical training units can negatively impact the emergence and course of MC. In animal experiments it could be proven that intensive exertion in mice infected with Coxsackie B3 led to a significantly increased mortality and more frequent pathological cardiac findings (myocardial fibrosis, ventricular dilatation) compared to animals without such physical training units 9 14 25 26 . In Swedish orienteers, the incidence of SCD was considerably reduced after a preventive training break was introduced for diseased athletes 27 .

#### Preventive training breaks

The question of whether and when a preventive training break is necessary can be difficult to answer in individual cases 3 14 . According to expert opinion, it is recommended that in cases of mild disease with symptoms from the neck upwards, such as a runny/blocked nose or a tickly throat, sport can be continued as long as the athlete feels physically fit enough 3 14 21 . The intensities should be within the regenerative range. Preferable would be a short training break even with a mild disease, or at least to shift the focus of the training to tactical/technical elements without cardiovascular exertion 3 .

Sport and competitive sport must be completely abandoned if the symptoms are below the neck or if systemic complaints occur, such as 3 14 :

- dyspnoea
- high temperature
- joint pains
- swollen lymph nodes
- gastrointestinal symptoms (e. g. diarrhoea)
- increased heart rate at rest
- severe cough.

The relevance of this training break should be made sufficiently clear to athletes since a high degree of motivation or a pressure to perform could tempt them to maintain or prematurely resume their training programme. Especially in the early days of the disease, the risk of pathophysiological changes is higher 3 . Once the symptoms have subsided, the break from training exertion should be upheld for at least 5–7 additional days, and resumption should start at a moderate level and gradually increase in intensity 3 14 .

### MC and COVID-19

According to the current literature, a SARS-CoV-2 (COVID-19) infection can also be concomitant with myocardial involvement 28 29 30 . In the early stages of the pandemic, the prevalence data were vague and reason to fear a high level of danger (e. g. very high prevalences of up to 78% 31 ). Meanwhile, many more studies have become available, and it is assumed that in approx. 1–3% of positively tested athletes, a myocardial involvement can be shown in the CMR 32 33 . A correlation between a positive finding and possible COVID-19 symptoms is not necessarily given. It is also conspicuous that a fair number of patients show pathological findings only in the CMR examination, whereas the ECG, TTE and troponin values frequently remain inconspicuous 32 . Long-term investigations to evaluate meaningfulness and prognosis are yet to become available.

#### Training break and diagnostics during/after COVID-19 infection

Different societies and authors have been fast to publish recommendations regarding when and with which precautionary measures it is viable to restart training and competitive sport after a COVID-19 infection 28 29 34 35 36 37 38 39 . Frequently (but not always), the severity of the COVID-19 symptoms is taken as a criterion for the duration of the preventive training break and the required screening instruments 38 40 41 . With an asymptomatic course, sport should be abandoned for between 7 and 14 days following a positive test. In symptomatic patients, sport should usually only be resumed at least 7–14 days after the symptoms have abated.

There is still no consensus among the societies regarding about which cardiological diagnostics are necessary prior to return-to-play (RTP). A compromise must be found between cost and benefit since the potential number of positively tested athletes would considerably exceed screening capacity (e. g. it is not practical to perform a CMR on every patient) 29 38 . Table 1 shows a comparison between different recommendations, especially for adult athletes in competitive sports. Usually basic diagnostics (e. g. medical history, physical examination, ECG) are recommended before RTP in conjunction with mild symptoms (sometimes also if asymptomatic). Depending on the findings, and with increasing severity and duration of the COVID-19 symptoms, more complex screening instruments can then also be added. Special RTP algorithms exist for athletes in competitive high school sports (<15 years) and for recreational master athletes (>65 years) 29 .

**Table:** **Table 1** Comparison of different return-to-play recommendations in patients/athletes after COVID-19 infection. If abnormal findings are detected, extended diagnostics are required. For detailed definitions and guidance, please see specific recommendations.

Recommendation	COVID-19 symptoms	Time before return-to-play	Recommended diagnostics before return-to-play
Nieß et al. 39 German Journal of Sport Medicine (May 2020)	Asymptomatic:	No intensive exercise for 2 weeks after positive test	Basic diagnostics: medical history and physical examination, laboratory tests and ECG
	Mild symptoms:	No exercise for 2–4 weeks after positive test	Basic diagnostics+extended diagnostics: stress ECG with O2 saturation, echocardiography, spirometry
	Severe symptoms:	No exercise for≥4 weeks after positive test	Basic diagnostics+extended diagnostics+CPET with BGA and body plethysmography
Phelan et al. 37 JAMA Cardiology (May 2020)	Asymptomatic:	No exercise for≥2 weeks after positive test	No specific cardiovascular risk stratification. If clinical and/or cardiac symptoms develop, follow appropriate clinical pathway
	Mild symptoms:	No exercise for≥2 weeks after symptom resolution	Clinical evaluation including 12 lead ECG+echocardiogram+laboratory test. Consider additional symptom-guided testing
	Severe symptoms:	No exercise for≥2 weeks after symptom resolution	Consider cardiac imaging per local hospital protocols. Consider repeated cardiac testing
Schellhorn et al. 36 European Heart Journal (May 2020)	Asymptomatic:	No intensive exercise for 2 weeks after positive test	ECG
	Symptomatic:	No exercise for≥2 to 4 weeks after positive test	Diagnostics according to severity. Cardiological follow-up (physical examination, resting+exercise ECG, echocardiography) after 2 to 4 weeks to get full sports release
Baggish et al. 35 British Journal of Sports Medicine (June 2020)	Asymptomatic:	N.A.	Focused medical history and physical examination. Consider 12-lead ECG. If ECG is abnormal, then additional evaluation with minimum echocardiogram and exercise test is warranted in conjunction with a sports cardiologist.
	Mild symptoms:	N.A.	Same as asymptomatic+ECG as mandatory
	Moderate to severe symptoms:	N.A.	Comprehensive evaluation prior return to sport, in conjunction with a sports cardiologist, to include blood biomarker assessment, 12-lead ECG, echocardiography, exercise testing and ambulatory rhythm monitoring
Kim et al. 29 JAMA Cardiology (October 2020)	Asymptomatic:	No exercise for 10 days after positive test	No specific cardiovascular risk stratification. If clinical and/or cardiac symptoms develop, follow appropriate clinical pathway
	Mild symptoms:	No exercise for 10 days from symptom onset (but must have full resolution of symptoms)	Specific cardiovascular risk stratification unnecessary, but on individual basis reasonable, particularly for protracted course of illness. If clinical and/or cardiac symptoms develop, follow appropriate clinical pathway
	Moderate symptoms:	No exercise for 10 days after symptom resolution	Medical evaluation+ECG+echocardiography+laboratory test. If abnormal: consider repeated cardiac testing+CMR+exercise test and extended ambulatory rhythm monitoring
	Severe symptoms:	No exercise for 14 days after symptom resolution	During hospitalisation: laboratory test+cardiac imaging
McKinney et al. 38 Canadian Journal of Cardiology (November 2020)	No evaluation stratified by COVID-19 symptoms	No≥moderate intensity exercise for≥7 days after complete viral symptom resolution; If cardiac symptoms are present: continued restriction from exercise	Focused cardiac symptom history. If cardiac symptoms are present after resolution of viral symptoms or a new unexplained reduction in fitness is present, then medical assessment is recommended, including history and physical examination and considering ECG and laboratory tests. In the presence of abnormal findings: referral to cardiology with advanced cardiac imaging (echocardiography and/or CMR) is recommended

Abbreviations: BGA, blood gas analysis; CMR, cardiovascular magnetic resonance imaging; CPET, cardiopulmonary exercise testing; ECG, electrocardiography; O2, oxygen.

#### RTP after COVID-19

RTP should be introduced with gradually increasing intensity. As a rule of thumb, 2–3 days of graduated return can be planned per training unit cancelled due to illness. This period should also serve to sufficiently regenerate the non-cardiac systems (e. g. pulmonary tissue, vasculature) 28 . In the training plan, first the frequency should be increased, then the duration and only finally the intensity 28 . Various graduated plans have been published and can be used for orientation 42 43 44 . In this context the following is important: as soon as cardiac symptoms (e. g. chest pain, palpitations) and/or an unexplained reduction in fitness occur, extended MC diagnostics are indicated 38 . Patients should be sensitised to this and be informed about the potential risk of SCD. In patients with ambiguous findings, the RTP strategy should be decided together with the athlete (shared decision-making) 29 45 . In contrast, in cases of confirmed MC the MC guidelines should be observed (see section RTP after MC).

#### MC after mRNA COVID-19 vaccination

Since mid-2021 there have been a growing number of reports that MC courses have been more frequently observed following a COVID-19 mRNA vaccination 46 47 48 . The mechanisms responsible for this are not yet fully understood 47 . Numbers from Israel show a higher incidence by factor 5.34 after BNT162b2 vaccination (Comirnaty, BionTech/Pfizer), compared with before the pandemic (factor 2.35 compared with an unvaccinated control group) 49 . The frequency of a vaccine-related MC is, however, lower than after contracting COVID-19 48 . Nevertheless, the risk was considerably increased following the second vaccination, especially in young men (factor 13.6 compared to pre-pandemic, factor 8.96 compared to unvaccinated control group) 49 . The observed course of the disease was luckily usually mild, with the symptoms emerging 2–5 days after the vaccination 47 49 50 51 . The German statistics also show a higher MC incidence rate following mRNA vaccination, particularly in young male patients 46 . The Spikevax vaccine (Moderna) proved to be the most risky and since mid-November has been recommended only for people above the age of 30.

There are currently no generally recognised restrictions regarding the interval between a COVID-19 vaccination and a return to sporting activity. It does appear reasonable, however, to refrain from exertion in the first few days, to await potential side-effects and to rest the body. The Ministry of Health in Singapore recommends refraining from exertive sport for at least 2 weeks following a COVID-19 vaccination 52 .

### Therapeutic options in conjunction with MC

In most cases, acute MC resolves favourably within a few weeks 20 . There are currently no controlled and randomised studies available for the optimised treatment of MC. Treatment involves a symptom-adjusted two-pillar approach, comprising 7 13 14 :

- treatment of heart failure in accordance with the guidelines, and
- treatment of arrhythmias in accordance with the guidelines.

In the case of acute symptoms, hospital admission and monitoring are necessary 14 . The results of an EMB are required, especially from patients in (pre-)cardiogenic shock or patients without long-term improvement in their symptoms, in order to give their treatment a specific direction 3 . Immunosuppressives are administered in cases of proven giant cell MC or sarcoidosis, while a specific antiviral therapy is commenced following a positive virus finding 3 5 .

For the treatment of advanced heart failure, the temporary use of mechanical circulatory support systems as a “bridge to recovery” can be necessary in a small percentage of patients 4 13 . Heart transplantation as the ultima ratio is not recommended until later, in order to allow time for potential recovery of the myocardium 20 .

In patients with arrhythmias, the danger of SCD can be minimised through the temporary use of a wearable cardioverter defibrillator (WCD). A permanent defibrillator system is implanted only later should symptoms persist 7 8 20 .

### Acute MC: training break and risk stratification

#### Training break

For patients with supposed or proven MC, the cardiological societies recommend a break from training and competitions of at least 3–6 months 6 10 . The recommendations are equally valid for hobby, amateur, and professional athletes, independent of their age, sex or global systolic function, and they can be lengthened depending on the disease course.

Prior to RTP, a comprehensive cardiological diagnosis is necessary for risk stratification. It includes the following examinations 3 6 7 10 :

- medical history/symptoms
- biomarkers and/or inflammatory markers
- TTE
- 24-hour Holter ECG
- stress TTE and/or cardiopulmonary exercise testing (CPET)
- CMR (recommended).

#### Risk stratification

MC patients are at permanent increased risk of malign arrhythmias due to possible myocardial scarring 6 8 14 . They also display a risk of insidious maladaptive remodelling of the myocardium, with a possible concomitant development of DCM 6 12 . A reliable test for whether the inflammation is still present or has subsided meanwhile is not currently available 2 6 . This is why current guidelines recommend annual check-ups (including ECG and TTE) for a period of at least 4 years, in order to observe the individual development 12 . Depending on the findings and the sporting ambition, the follow-up observation phase can be significantly extended.

For risk stratification, evaluation of the LVEF and increasingly also the change of a possible positive LGE finding are used. In asymptomatic patients with an uncomplicated disease course, preserved systolic function and a negative LGE finding, the prognosis is very good 3 15 . In a study with a total of 670 suspected MC cases 15 , the annual event rate in the first 5 years following onset of disease in patients with an LVEF>40% and without an LGE finding was 1.1% for MACE and 0.4% for mortality. In cases of a positive LGE finding (LVEF still>40%), the annual event rates rose to 2.6 and 1.2%, respectively.

An isolated reduced LVEF was accompanied by a poorer prognosis (MACE: 6.4%; mortality 2.8%). The highest annual event rates were observed in patients with restricted LVEF (<40%) and positive LGE finding (MACE: 10.5%; mortality: 3.1%) 15 .

The isolated interpretation and weighting of a positive LGE finding is currently still being intensively researched and discussed 15 16 .

### RTP after MC

The precise scheduling of permitted resumption of sporting activity has to be decided from case to case and in discussion with the patient 3 7 . In the acute phase of MC, strict physical rest is indicated 53 54 55 . The relevance of the sports break and the risks of not adhering to it should be expressly conveyed.

According to the current recommendations, a release for intensive sporting activities and competitions can be issued 3–6 months after the acute disease phase, provided that the following criteria are observed 6 10 :

- ventricular systolic function within normal range
- cardiac and inflammatory biomarkers within normal range
- lack of clinically relevant arrhythmias in daily routines (24-hour Holter ECG) and in stress situations (e. g. CPET).

The importance of re-assessments should be pointed out once again 6 12 .

Two recent overviews provide additional pointers for a more differentiated evaluation 3 7 : constitutive physical training should be started at the earliest 1 month after the acute disease phase, and only with light and moderate intensities (no high-intensity interval training, HIIT), e. g. within the framework of cardiac rehabilitation 3 53 55 . In stable patients with an uncomplicated course, inconspicuous cardiac and inflammatory biomarkers, preserved systolic function and no positive LGE indication, exertion levels can be increased successively after 3 months into the intensive range 3 7 in conjunction with prognostically low complication rates 56 .

If during the acute phase of MC, in contrast, there was a restricted LVEF and/or positive LGE indication of LGE, intensive physical exertion (e. g. HIIT) and competitive sport should be avoided for at least 6 months, even if the systolic function has recovered in the meantime 3 .

In MC patients with restored systolic function after 6 months yet still displaying a positive (albeit not worse) LGE indication, there is a theoretically an increased risk of ventricular arrhythmias linked to potential SCD. In such cases, a release to sport can be granted only after thoroughly informing the patient 3 6 7 . Shared decision-making between the clinician and patient is encouraged, accompanied by annual routine check-ups for long-term risk stratification 3 6 14 .

A release to intensive sports and competitions cannot be granted if a lowered LVEF or other indications of incomplete myocardial recovery are still present after 6 months 3 . In such cases, a re-evaluation should take place at 3- to 6-month intervals.

In cases where there is no improvement, not even in the long term, a decision must be reached for the individual patient and together with that patient. The decision process should include the nature of the envisaged sport with regard to its cardiovascular impact (e. g. golf vs. football), as well as the risks involved in losing consciousness (e. g. during motor sports or diving) 3 . In some circumstances, recommendations could extend to complete abstinence from extreme exertion and competitive sport in the future 3 17 57 . Moderate regular training at “rehabilitation level” is, however, possible and also advisable 3 .

#### Training design

In the training design a difference is made between healing and healed MC 58 . In the healing phase, training may take place only in conjunction with a stable clinical status and significantly abating symptoms (e. g. 24-hour Holter ECG, TTE, biomarkers) 53 55 . A maximal exercise test is still counter-indicated at this stage and the training units should only have very low intensities (values on rate of perceived exertion (RPE): 6–8, scale: 6–20) 53 55 58 .

The training design following healed MC draws upon many years of experience with heart failure patients, as well as the results of the individual symptom-limited exercise test 7 53 55 59 60 . In the first 4–6 weeks, the training should be performed with low to moderate intensity (no maximum effort) to approx. 40–50% of the maximal oxygen uptake (peak VO _2_ ) (RPE:<10–12) 3 7 53 59 60 . Should conspicuities occur (e. g. clinically relevant arrhythmias), training must be stopped immediately 53 58 . If the patients tolerate the exertion well, more intensive units to approx. 50–60% (up to<75%) of peak VO _2_ (RPE: 12–14) can be performed over the course 58 . After approx. 6 and 12 weeks, as well as before the implementation of highly intensive units (e. g. HIIT), a new maximal exercise test should exclude possible conspicuities 3 .

A moderate dynamic resistance training should start with low exertion (30–50% of the 1 repetition maximum (1-RM), RPE: 12–13), with the intensity increasing gradually over the course (40–60% of the 1-RM, RPE: 14–15) 53 59 60 . If intensive physical exertion is tolerated well, and no other findings contradict it, a release for competitive sport can be given in the long term 3 .

#### Education and mental health

Adequate education of patients and their coaches or training partners is a crucial aspect for reliable training design. Experience has shown that ideas about moderate/regenerative training plans can diverge considerably, and that consistent compliance is unfortunately not a given in all cases 3 57 . Against this background, the recommended training ranges and intensities should be clearly communicated and the information given should be documented in writing, also in order to circumvent later liability issues 3 .

With regard to emergency prophylaxis, it is vital to instruct and educate those with direct access to the patient/athlete since first aid is crucial if a haemodynamically relevant arrhythmia should occur. Training in groups, knowledge about adequate first aid measures, and the rapid availability of an automated external defibrillator (AED) have all proven favourable for the prognosis 1 61 . Education can and should therefore include the following aspects:

- training range and intensity during the first post-MC phase
- possible consequences if ignored
- symptoms of possible complications
- first aid measures
- relevance of regular check-ups
- prevention of renewed MC.

Another significant point for RTP is the mental health of the patient: for ambitious amateur or professional athletes, an uncertain long-term medical prognosis, long training breaks, a lack of competitions and/or the cancellation of sponsoring/prize money can represent a huge psychological burden and tempt athletes to resume intensive physical activities too soon. Potential anxieties can also restrict RTP following resolved MC. The early integration of a psychological carer should therefore be considered in relevant constellations 3 .

## Summary and outlook

MC is a frequent cause of malignant arrhythmias and SCD in young, active, physically fit persons with no further pre-existing conditions 1 . Due to a heterogeneous course, its diagnosis is complex, and diseases often remain undiscovered 3 4 17 . A predominantly reliable diagnosis can be achieved only by combining various techniques, such as medical history, ECG, TTE, laboratory testing, CMR and, in individual cases, EMB 3 . It should be noted that atypical changes – especially in athletes – do not always have to be pathological (cf. athlete‘s heart) 17 .

**Fig. 1:**
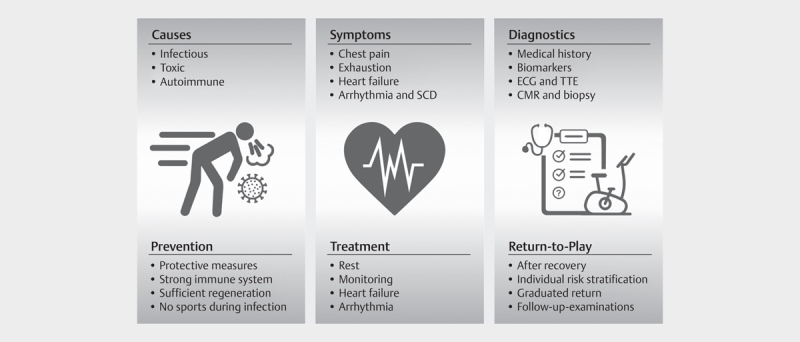
Key components of myocarditis and sports. Abbreviations: SCD, sudden cardiac death; ECG, electrocardiography; TTE, transthoracic echocardiography; CMR, cardiovascular magnetic resonance imaging.

In most cases, acute MC resolves. Depending on the disease course, however, some patients have a long-term increased risk of prognostically relevant arrhythmias and/or progressive maladaptive remodelling up to DCM 8 . Both in the prevention and in the therapy of MC, temporary abstinence from intensive sporting activities plays a crucial role and acquires additional relevance within the context of the COVID-19 pandemic. Following recovery from MC, training should take place only at moderate intensity. Over the course, monitored maximal exercise tests and routine follow-up diagnostics permit individual risk stratification and can – depending on the findings – lead to release for intensive sporting activities and competitive sport 3 6 7 10 **(** Fig. 1 **)** .

In the future, multi-centre registry studies in combination with the latest diagnostic options are desirable 3 7 . In order to hinder the emergence of MC through preventive training breaks, augmented health education (e. g. for amateur athletes, professional athletes, coaches, teachers) should be a goal. In addition, first aid courses can considerably improve the quality of immediate care and reduce the incidence of SCD during sporting activities 1 61 .
